# Edible wild plants, chicory and purslane, alleviated diabetic testicular dysfunction, and insulin resistance via suppression 8OHdg and oxidative stress in rats

**DOI:** 10.1371/journal.pone.0301454

**Published:** 2024-04-11

**Authors:** Enas A. Saad, Hanaa A. Hassan, Mamdooh H. Ghoneum, Mai Alaa El-Dein

**Affiliations:** 1 Zoology Department, Faculty of Science, Mansoura University, Mansoura, Egypt; 2 Department of Surgery, Charles Drew University of Medicine and Science, Los Angeles, CA, United States of America; 3 Department of Surgery, University of California Los Angeles, Los Angeles, CA, United States of America; Alexandria University, EGYPT

## Abstract

Testicular dysfunction is a prevalent health problem frequently reported in individuals with diabetes mellitus (DM). Oxidative-inflammatory reactions, hormonal and spermatic abnormalities often accompany this illness. Herbal remedies “particularly wild plants” including chicory (*Chicorium Intybus)* and purslane (*Portulaca Oleracea)* are emerging as popular agents for people dealing with these issues due to their ability to act as antioxidants, reduce inflammation, and exhibit antidiabetic effects. According to the collected data, the daily administration of chicory (Ch) seed-extract (250 mg/kg) or purslane (Pu) seed-extract (200 mg/kg) to streptozotocin (STZ)-induced diabetic rats (50 mg/kg) for 30 days resulted in the normalization of fasting blood glucose (FBG), serum fructosamine, insulin levels, and insulin resistance (HOMA-IR), as well as reducing lipid peroxidation end-product malondialdehyde (MDA) level, aldehyde oxidase (AO) and xanthene oxidase (XO) activities. While caused a considerable improvement in glutathione (GSH) content, superoxide dismutase (SOD), catalase (CAT) activity, and total antioxidant capacity (TAC) when compared to diabetic rats. Ch and Pu extracts had a substantial impact on testicular parameters including sperm characterization, testosterone level, vimentin expression along with improvements in body and testis weight. They also mitigated hyperlipidemia by reducing total lipids (TL), total cholesterol (TC) levels, and low-density lipoprotein cholesterol (LDL-C), while increasing high-density lipoprotein cholesterol (HDL-C). Furthermore, oral administration of either Ch or Pu notably attuned the elevated proinflammatory cytokines as tumor necrotic factor (TNF-α), C-reactive protein (CRP), and Interleukin-6 (IL-6) together with reducing apoptosis and DNA damage. This was achieved through the suppression of DNA-fragmentation marker 8OHdG, triggering of caspase-3 immuno-expression, and elevation of Bcl-2 protein. The histological studies provided evidence supporting the preventive effects of Ch and Pu against DM-induced testicular dysfunction. In conclusion, Ch and Pu seed-extracts mitigate testicular impairment during DM due to their antihyperglycemic, antilipidemic, antioxidant, anti-inflammatory, and antiapoptotic properties.

## Introduction

Hyperglycemia, a complicated metabolic disease associated with DM, frequently results from issues with insulin secretion or metabolism, or perhaps both. As a result of its chronic nature, patients’ quality of life suffers, and healthcare expenses rise [1]. According to the systematic analysis for the Global Burden of Disease Study 2021, By 2050, more than 1.31 billion (1.22–1. 39) people are projected to have diabetes 2050 [2]. DM exerts a substantial impact on male fertility, through both direct and indirect mechanisms [3]. The incidence of several complications associated with DM, including reproductive dysfunction, can be attributed to oxidative stress [4]. DM has the potential to elicit structural alterations in the testes and spermatozoa, leading to germ cell death, impaired sperm characteristics, and hormonal imbalances, ultimately culminating in male infertility [5]. The occurrence of male reproductive modifications has been extensively seen in both experimental animal models and humans with DM [6]. The induction of DM in male rats through the administration of Streptozotocin (STZ) has been observed to result in the atrophy of the sex organ and abnormalities in the histoarchitecture of the prostate [7] and decreased sperm count [8] as well as testosterone level [9], these impairments were accompanied with elevated serum levels of lipids, including triglycerides and cholesterol [10].

One of the primary contributors to male infertility is oxidative stress [11, 12]. Insufficient endogenous testicular antioxidants pose a challenge in safeguarding the maturation of germ cells against an excessive presence of reactive oxygen species (ROS) induced oxidative damage. Excess ROS has the potential to induce oxidative harm to cellular constituents, including lipids, proteins, and nucleic acids (DNA, RNA) [11, 12].

Elevated ROS levels are associated with peroxidative damage to the spermatozoal membrane, which contains a high concentration of docosahexaenoic acid [13], this results in an elevated permeability of the sperm membrane, causing morphological irregularities that ultimately lead to the development of severe oligospermia, asthenospermia, and male infertility [14]. Multiple studies have documented that persons with DM experience an increase in proinflammatory cytokines in their serum as a result of hyperglycemia [15], these trigger pancreatic β-cells apoptosis by the activation of the caspase family cascade [16]. Furthermore, an increase in ROS is linked to damage in mitochondrial DNA, resulting in the initiation of DNA damage response. This process involves the overexpression of proapoptotic Bax and the downregulation of antiapoptotic Bcl-2 in the testes of rats with diabetes [17]. Prior research has documented an elevated concentration of 8-hydroxy-2-deoxyguanosine (8-OHdG), which is recognized as one of the most prevalent types of oxidized DNA, in individuals with chronic diseases, including DM [18]. Consequently, oxidative damage may lead to impaired testis’s function, decreased sperm parameters, and ultimately, male infertility [19, 20].

Vimentin serves as an intermediate filament and functions as a cytoskeletal protein in Sertoli cells (SCs) and Leydig cells. The degree of vimentin expression is directly associated with the maintenance of the morphological integrity of the seminiferous epithelium [21]. The aberrant expression of vimentin may compromise its functionality and promote apoptosis in spermatogenic cells [22]. Previous studies have demonstrated that the downregulation of vimentin leads to the disruption of structural integrity and functional impairment of Sertoli cells in the testes of diabetic rats [23].

Currently, the existing synthetic antidiabetic medications are associated with significant adverse effects [24]. Hence, the World Health Organization advocates for conducting study on the advantageous utilization of medicinal herbs in the therapy of DM [25]. The efficacy of natural plants is mostly attributed to the presence of bioactive compounds, including flavonoids, polyphenols, tannins, chalcones, and carotenoids [26]. Ch and Pu are Egyptian edible wild plants that are classified under the botanical families *Asteraceae* and *Portulacaceae*, respectively [27–29]. These plants are known for their high content of antioxidants and flavonoids, which have been found to facilitate the removal of pollutants and mitigate oxidative stress. Additionally, they have been seen to protect against testicular injury through several mechanisms [30–32].

All morphological parts of Ch contain a great number of various chemical compounds [33]. Ch leaves, roots and seeds are known to possess several nutrients and bioactive chemicals, including inulin, vitamins A, B1, B2, and C, as well as calcium (Ca), magnesium (Mg), sodium (Na), potassium (K), iron (Fe), copper (Cu), manganese (Mn), zinc (Zn), and phenolic compounds, among other constituents [34]. Numerous extracts derived from Ch have exhibited a diverse array of biological and pharmacological attributes, including antidiabetic, anti-inflammatory, and antioxidant activities [35].

Pu is an herbaceous plant that thrives in warm climates. It is an antioxidant agent which is useful in providing nourishment and enhancing the activity of antioxidant enzymes in hepatic, renal, and testicular tissues [36–38]. Its leaves and seeds are known for their abundant content of dietary antioxidants, encompassing flavonoids, glutathione, omega-3 fatty acids, alkaloids, and vitamins, alongside dietary minerals [39]. The secondary metabolites present in Ch and Pu have demonstrated robust antioxidant, metal-chelating, and anti-inflammatory properties, which have beneficial effects on the health of both humans and livestock [40]. Both Ch and Pu extracts’ neutralizing free radicals’ effects can prevent the adverse effect of ROS on spermatogenesis, protect the sperm, and improve the male reproductive function [41, 42].

The present study was designed to evaluate the prophylactic role of Ch and Pu seed extracts against testicular dysfunction associated with STZ-induced diabetes in rats.

## Materials and methods

### Preparation of ethanolic seed extracts of both chicory and purslane

Seeds of Ch and Pu were purchased from local markets of Mansoura city, Egypt. Mansoura University’s Unit of Genetic Engineering and Biotechnology at the Faculty of Science was responsible for the authentication and processing of the seeds. To ensure full drying, before being ground, seeds were heated to 40 ºC for 12 h. A total of 60 ml of ethanol (80%) was poured into 20 g of ground seeds from each plant, and the conical flasks were placed on a horizontal water bath shaker set to 45°C and 200 rpm for 4 h. After two days of incubation at 45°C, the mixture was filtered through Whatman filter paper and a Buchner funnel. The obtained extract in each case was dried at 45°C until complete dryness and then weighed. For Pu, 20 g of dried extract was dissolved in 1,000 ml of distilled water, while for chicory, 25 grammes of dried extract was dissolved in 1,000 milliliters of distilled water. The solutions were well shaken until complete dissolving of the dry weight of the extracts. The total yield % of the extracts was about 2.457% (w/v) for Ch and 1.146% (w/v) for Pu. Rats were administered the prepared extracts orally at a dose of 250 mg/kg BW/ml for Ch and 200 mg/kg BW/ml for Pu for a period of four weeks.

### Phytochemical analysis of the seed extracts

Ch and Pu phenolic content was determined using the Folin-Ciocalteu (F-C) test, with characteristic results calculated as mg Gallic acid equivalents/g of the dried plant material using a Gallic acid standard curve (y = 0.0062x, r^2^ = 0.987) [43]. Using an aluminum chloride colorimetric technique, the flavonoid content of Ch and Pu seed extracts is expressed as mg catechin equivalent per g of dry plant material [44], using the standard curve of Catechin (y = 0.0028 x, r^2^ = 0.988). The vanillin hydrochloride assay method was used to determine the tannin concentration in Ch and Pu seed extracts [45], after exposing the samples to freshly made vanillin-hydrochloride, the absorbance was measured. The measured tannin contents were expressed in terms of tannic acid equivalent mg per g of dry plant material. Using the standard curve for tannic acid (y = 0.0009x; r2 = 0.955).

### Experimental animals

Thirty-six Adult male normoglycemic Wistar rats (*Rattus Rattus*) weighing 120 ± 10 g at 4–5 weeks of age, were used in this present study. Rats were purchased from the Egyptian Vaccine Company (VACERA, Cairo, Egypt). At the Animal House Lab., Faculty of Science, Mansoura University, Dakahlia, Egypt, animals were housed in stainless steel cages and kept in a temperature-controlled room between 22 and 25°Cwith free access to food and water. They were also kept in a pathogen-free environment with a 12-h light/dark cycle. Before putting them through any kind of test, they were allowed to acclimate to the laboratory environment for one week in order to adapt.

### Ethical approval

The animals received appropriate care, medical treatment, and euthanasia in compliance with the guidelines set by the National Institute of Health (NIH) for the care and use of laboratory animals., All procedures were approved by the Animal care and use committee in the College of Science at Mansoura University in Dakahlia, Egypt. Code number: **MU-ACUC (SCMS.22.10.5)**.

### Establishing the experimental DM model

After acclimatization, DM was induced by feeding animals on a high-fat diet (HFD) consisting of (3.4 kcal/g with 24.8% w/w fat, 54.6% w/w carbohydrate and 12.8% w/w protein) containing 60% fat calories was formulated by combining butter (7.4 kcal/g with 55% saturated fat). After 2 weeks of HDF, animals were fastened overnight and injected intraperitoneally with STZ (50 mg/kg) which was purchased from (Sigma Aldrich® Company, USA), STZ was freshly prepared by dissolving in citrate buffer (0.1M, pH:4.5) [46, 47]. Diabetes induction was confirmed by analyzing serum glucose levels using a glucometer 3 days after STZ injection. The diagnosis of diabetes was confirmed when the rat’s fasting blood glucose level exceeded 200 mg/dl.

### Animal grouping

Animal were distributed into 6 groups (n = 6 per group) as: **Control rats group (C),** healthy rats were fed on a standard laboratory diet consisting of a fat source in the (2.4 kcal/g with 6% w/w fat, 68.2% w/w carbohydrate and 16% w/w protein) and free access to running water; **Chicory rats group (Ch),** normal rats fed on a standard laboratory diet and orally received 250 mg/Kg [48] of 80% ethanolic Ch seed extract daily for 30 days; **Purslane rats group (Pu),** normal rats fed on a standard laboratory diet and orally received 200 mg/Kg [49] of 80% ethanolic Pu seed extract daily for 30 days; **Diabetic rats group (STZ),** rats were fed on HFD two weeks before the intraperitoneal injection of STZ 50 mg/Kg in a single dose to induce experimental DM, and fed on HFD for 30 days; **Diabetic and chicory rats group (STZ+Ch),** rats were fed on HFD two weeks before the intraperitoneal injection of STZ 50 mg/Kg in a single dose and orally administered with 250 mg/Kg with ethanolic seed extract 80% of Ch daily for 30 days; **Diabetic and purslane rats group (STZ+Pu),** rats were fed on HFD two weeks before the intraperitoneal injection of STZ 50 mg/Kg in a single dose and orally administered with 200 mg/Kg with ethanolic seed extract 80% of Pu daily for 30 days. Throughout the experiment duration, the animals’ body weights were recorded weekly, and the subsequent weight gain was calculated.

### Sera and tissue sampling

At the end of the experiment, rats were fastened overnight then sacrificed under ketamine/xylazine (0.1 ml/100 g) intraperitoneally anesthesia. Blood samples were collected via tail vein for FBG level, and via cardiac puncture for the rest of biochemical analysis in sterile centrifuge tubes [47], let to clot at room temperature for 15 minutes before centrifuging them at 3000 rpm for 15 minutes at 4°C. Serum glucose level was immediately measured, then the remaining sera were labeled and frozen quickly at -20°C for biochemical analysis. Testicular tissue was carefully dissected out from each animal and weighed. For subsequent histopathological and Immunohistochemical staining, right testes were preserved in neutral formalin (10%). While the left ones were homogenized in a teflon homogenizer with ice-cold normal saline solution to yield a 10% (w/v) homogenate, the homogenates were centrifuged at speed of 3000 rpm for 20 min, then frozen at -20°C for additional biochemical and antioxidant analysis. Caudal epididymis from each animal was dissected transferred into sterile bottle containing 3 ml of normal saline for semen analysis to estimate count and motility of sperms.

### Biochemical parameters

Kits from (Biodiagnostic Egypt; Catalogue #: GL 13 20) and (Biovendor RandD, Czech Republic; Catalogue #: RTC018R) were used to measure serum glucose and insulin concentrations, respectively. The kit (Assaygenie, Duplin, Ireland, London; Catalogue #: RTEB1805) was used to determine fructosamine levels. Following this approach, we were able to determine the homeostasis model of insulin resistance (HOMA-IR) using the following formula:

HOMA-IR=(FBG×Fastinginsulin)/405

Malondialdehyde (MDA) is estimated in testis homogenate using kits supplied from (Bio-diagnostic, Egypt, Catalogue # MD 25 29). The levels of Aldehyde oxidase (AO) and Xanthene oxidase (XO) in in testis tissues were evaluated using ELIZA kits provided by Aviva system biology (San Diego, USA, Catalogue # OKEH03524) and Cusabio (Texas, USA, Catalogue # Catalog CSB-E13614r), respectively. Antioxidant enzymes (superoxide dismutase, SOD, Catalogue # SD 25 21), catalase (CAT, Catalogue # CA 25 17), total antioxidant capacity (TAC, Catalogue # TA 25 13), and glutathione concentrations (GSH, Catalogue # MBS545286) were measured in the testis as directed by the manufacturer, (Bio-diagnostic, Egypt). Serum testosterone concentrations were measured using an ELISA kit purchased from My Biosource (San Diego, USA; Catalogue # MBS282195).

Total lipids (TP), Total cholesterol (TC), triglyceride (TG) and high-density lipoprotein cholesterol (HDL-C) were measured using commercially available test kits (Biodiagnostic, Egypt, Catalogue #: TL 20 10, TC 12 20, TG 20 30, CH 12 30) respectively. Friedewald’s formula [50] was used to calculate low-density lipoprotein cholesterol (LDL-C) as follow:

LDL-C=TC–TG/5–HDL-C=mg/dl

Serum IL-6 levels were measured using commercially available Quantikine® ELISA (Minneapolis, USA; Catalogue #: R6000B) kits. Kits (BD-bioscience, San Jose, USA, Catalogue #: 557825) are used to measure C-reactive protein (CRP). For the purpose of approximating tumor necrosis factor alpha (TNF-), we used (My Biosource, Southern California, San Diego, USA, Catalogue #: MBS175904). Additionally, 8-hydroxy-2-deoxyguanosine (8-OHdG) levels in testes were measured using an ELISA kit from Cusabio (San Diego, USA, Catalogue #CSB-E10526r). ELIZA kits for evaluating testis Bcl-2 were available from My Biosource (San Diego, USA; Catalogue #: MBS2515143 96T).

### Histopathological and immunohistochemical examination

After being removed from the animals, the testicular tissues were fixed in Bouin’s solution for 6 h and then stored in 70% ethanol. After being fixed in a graded ethanol series, the tissues were dehydrated, cleaned in xylene, and vertically embedded in paraffin. Rotor microtomes were used to cut sections that were 4 μm thick. Hematoxylin and eosin (H&E) staining was performed on paraffin sections that had been dewaxed in xylene for 20 minutes and then rehydrated in a series of progressively more dilute ethanol solutions.

Johnsen’s testicular score system was used to quantify the degree of histological damage to testicular tissue. Each group had 30 cross-sectioned tubules assessed, and using Johnsen’s criteria, each tubule was given a score between 1 (very bad) and 10 (excellent) [51].

For immunohistochemistry, deparaffinized and hydrated testis slices were washed with PBS for 15 min, then treated for 10 min in 0.3% hydrogen peroxide to suppress endogenous peroxidase activity. It was done using the labelled streptavidin-biotin immunoperoxidase method. The rabbit polyclonal primary antibodies for caspase-3 (ab13847), and mouse primary antibodies anti-vimentin (a Sertoli cell marker, 1:50 mice anti-rat, sc-6260; Santa Cruz Biotechnology, Inc, Dallas, TX) were used. Primary antibodies were incubated on sections over the course of one night at 4°C; following that, image J software was used to assess the labelled index.

### Statistical analysis

GraphPad prism 9.0 software (GraphPad prism software Inc., San Diego, California, USA) was used to statistically analyze all of the data. Results are presented as mean ± standard error of the mean (SEM) of n = 6. One-way analysis of variance (ANOVA) was used to analyze statistical comparisons, followed by the Tukey test. In comparison to the control and STZ groups, the percentage of changes in the treatment groups was computed, a difference was deemed significant when the *P* value was less than 0.05 and any higher significance level was mentioned. The following symbols of significance level were used to denote statistical differences: *P < 0.05, **P < 0.01, ***P < 0.001 and ****P < 0.0001 vs. control group. ^@^*P* < 0.05, ^@@^*P* < 0.01, ^@@@^*P* < 0.001 and ^@@@@^*P* < 0.0001 vs. STZ group.

## Results

### The phytochemical analysis of seed extracts

The amount of phenolic compounds was expressed as (mg gallic acid / 1 g dry extract), while (mg catechin / 1 g dry extract) was used to express the flavonoid content, and the tannin content was expressed as (mg tannic acid / 1 g dry extract) based on the data supplied Table 1.

**Table 1:** Phytochemical analysis of seed extracts.

Phytochemical Analysis	Ch	Pu
**Phenolic Content (mg /g)**	85.04	45.60
**Flavonoid Content (mg /g)**	58.59	29.19
**Tannin Content (mg /g)**	5.13	3.34

### Ch and Pu enhanced body weight gain and testicular efficiency

**Fig 1:**
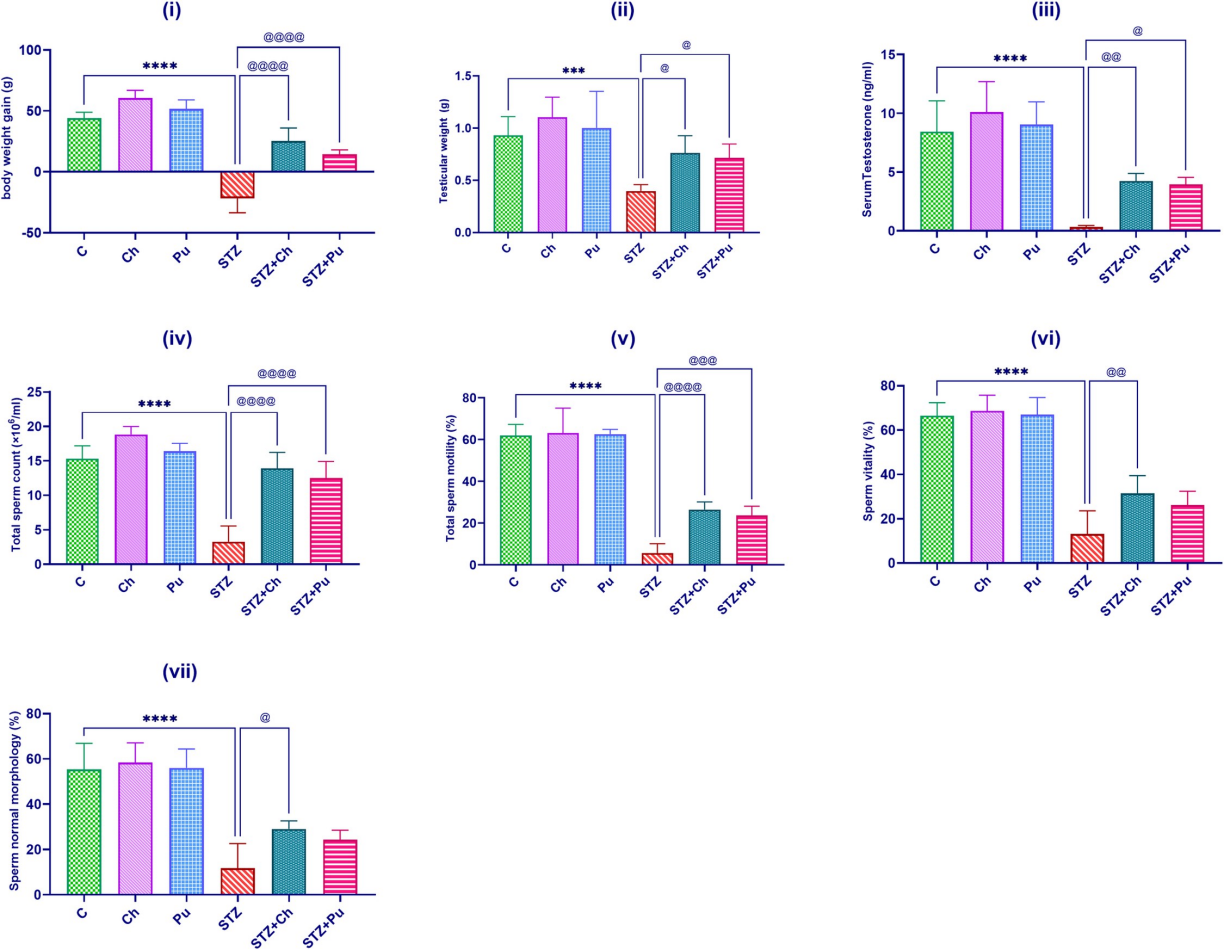
Effect of STZ, Ch and Pu on: (i) Body weight gain; (ii) Testicular weight, (iii) Serum Testosterone level; (iv) Total sperm count; (v) Total sperm motility; (vi) Sperm vitality; (vii) % of Sperm normal morphology of rats in the different groups. Values are expressed as means ± SEM; (n = 6). (***P<0.001 and ****P< 0.0001 vs. control group. ^@^*P*<0.05, ^@@^ *P*<0.01, ^@@@^ *P*<0.001, ^@@@@^ *P*<0.0001 vs. STZ group).

Administration of Ch and Pu seed extract to normal rats revealed a statistically insignificant enhancement in both body weight gain and testicular weight with a comparable result to the control rats (+37.3%, + 17.5% for the body weight gain) and (+18.6%, +7.7% for testicular weight) respectively with respect to normal control group. While a notable decline in body weight gain -149.7%, *P*<0.0001, together with testicular weight -57.3%, *P*<0.001 was recorded in untreated diabetic rats comparing to control group. Intriguingly, treating diabetic animals with Ch or Pu significantly attuned body weight gain (+115.5% and +65.8%, *P*<0.0001 respectively) as well as testis weight (+91.7% and +79.7%, *P*<0.05 successively) regarding to the STZ group. Serum testosterone levels were observed to be considerably lower in the diabetic group compared to controls (-96.3%, *P*<0.0001) and were significantly rectified toward control levels after treatment with Ch (+1265.6%, *P*<0.01), and Pu (+1174.2%, *P*<0.05) with respect to STZ group as shown in Fig 1.

Observed results (Fig 1) didn’t show any notable changes in total sperm count, motility, vitality, and morphology by administration of either Ch or Pu seed extracts to normal rats when compared with the control group. In contrast, the STZ group exhibited a substantial reduction in total sperm count, motility, vitality, and morphology compared to the control group (-78.7%, -90.9%, -80.2%, -78.9%, *P*<0.0001). Meantime, the daily administration of either Ch or Pu seed extracts to diabetic animals significantly ameliorated: total sperm count (+325.5% and +282.7%, *P*<0.0001); sperm motility (+367.6, *P*<0.0001 and +317.7%, *P*<0.001); sperm vitality (+139.2%, *P*<0.01 and +98.7%); and sperm morphology (+148.6%, *P*<0.05 and +108.6%) respectively versus untreated STZ rats group.

### Ch and Pu seed extract attuned hyperglycemic profile

**Fig 2:**
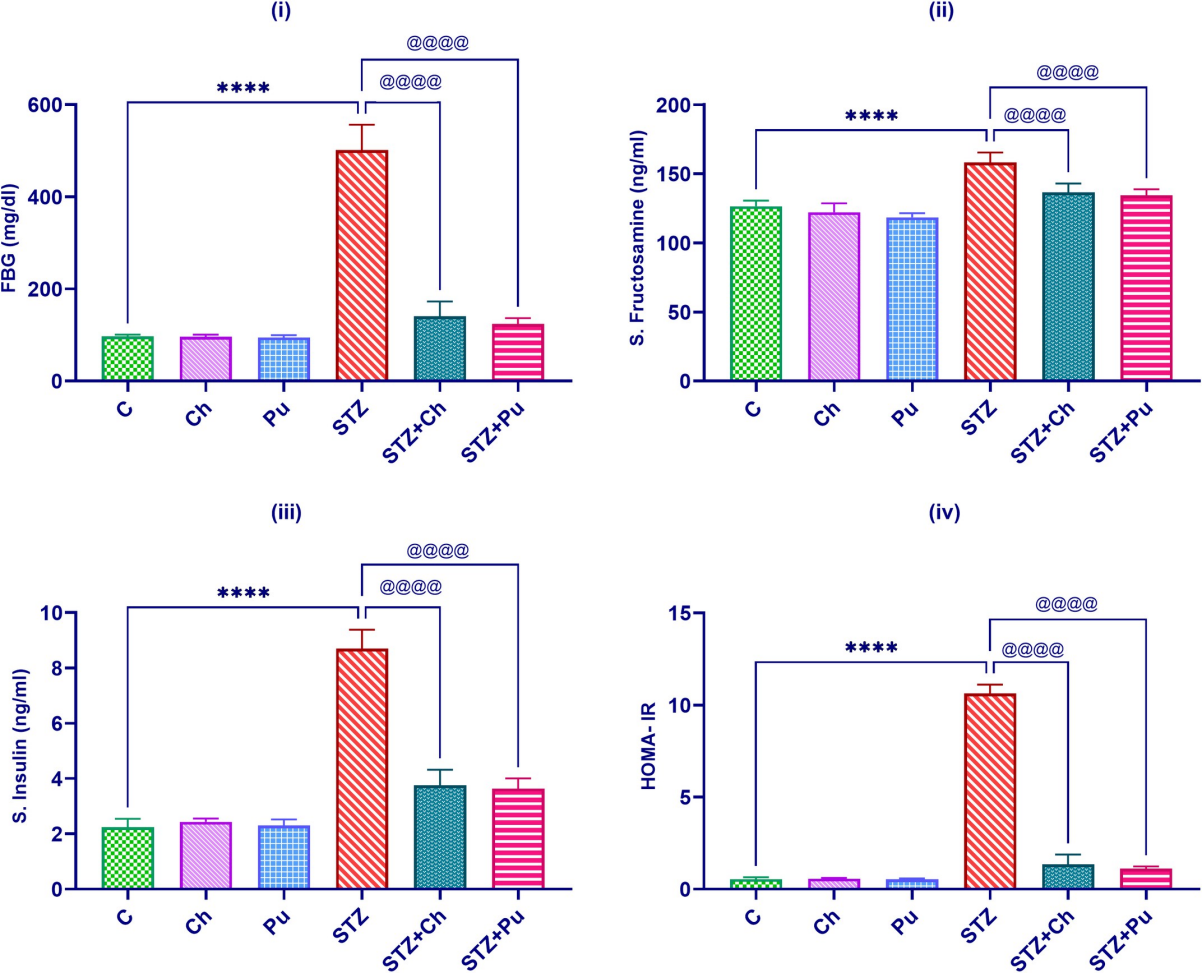
Effect of STZ, Ch and Pu on: (i) FBG (mg/dl); (ii) Serum fructose amine (ng/ml); (iii) serum insulin (ng/ml); (iv) HOMA-IR % of rats in the different groups. Values are expressed as means ± SEM; (n = 6). (****P<0.0001 vs. control group. ^@@@@^*P*< 0.0001 vs. STZ group).

At the end of the experiment, a significant elevation in FBG and fructose amine levels (*P*<0.0001) was observed in untreated STZ animals, the blood glucose level being about 501.5 mg/dL, while fructose amine was 158.2 ng/ml. Treatment of diabetic animals with Ch and Pu for 30 days resulted in a significant (*P*<0.0001) lowering in both FBG level (-72% and -75.3% respectively) and fructose amine (-13.7% and -14.9% respectively) when compared to untreated STZ group. Assessment of Serum insulin level and HOMA-IR revealed a remarkable elevation in the STZ group (*P*<0.0001) comparing to the normal group. Administration of Ch seed extract to diabetic animals significantly attuned both serum insulin level and HOMA-IR percentage by -56.9% and -87.3% (*P*<0.0001) respectively with respect to untreated STZ group. Furthermore, administration of Pu seed extract to diabetic rats markedly reduced serum insulin level and HOMA-IR percentage near to their normal levels (-58.2% and -89.5%, *P*<0.0001) respectively, with respect to untreated STZ group (Fig 2).

### Ch and Pu seed extract regulated the lipid profile

**Fig 3:**
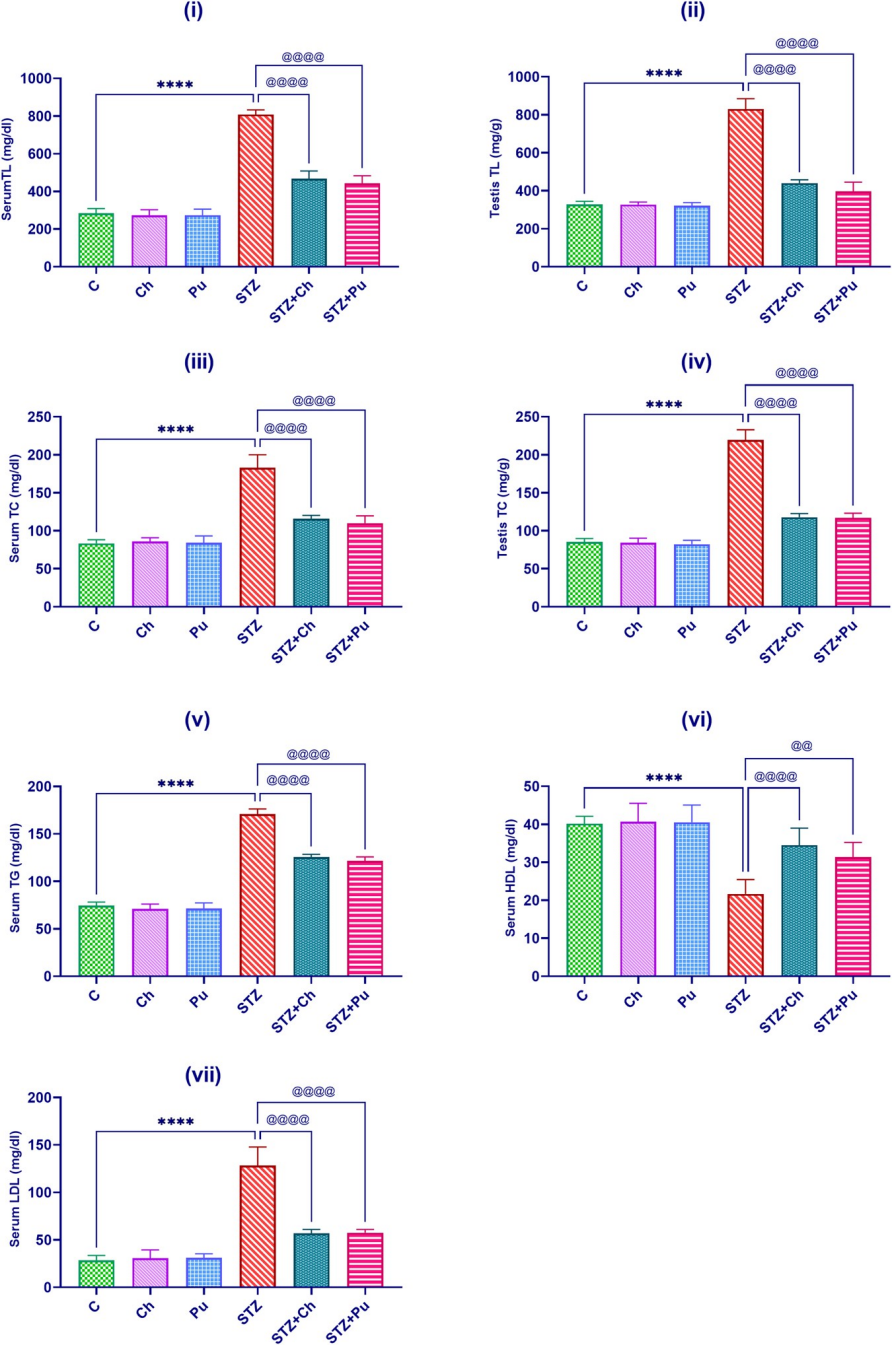
Effect of STZ, Ch and Pu on: (i) Serum TL (mg/dl); (ii) Testis TL (mg/g), (iii) Serum TC (mg/dl); (iv) Testis TC (mg/g); (v) Serum TG (mg/dl); (vi) Serum HDL (mg/dl); (vii) Serum LDL (mg/dl) in the different experimental groups. Values are expressed as means ± SEM; (n = 6). (****P < 0.0001 vs. control group. ^@@^*P*< 0.01 and ^@@@@^ *P* < 0.0001 vs. STZ group).

Fig 3 indicated a remarkable increase in both serum and testicular TL (+184.9%, +152.7%, *P*<0.0001); and TC (+119.4%, +157.7%, *P*<0.0001) levels, along with serum TG and LDL (+128.9%, +353.2%, *P*<0.0001, respectively), Meantime a significant decrease (-46.1%, p<0.0001) in serum HDL level was noticed in STZ rats versus control.

Diabetic rat administered Ch and Pu seed extracts showed marked decrease in serum TL (-42.2% for Ch, and -45.2% for Pu, *P*<0.0001); testis TL (-46.5% for Ch, and -52.5% for Pu, *P*<0.0001); serum TC (-36.5% for Ch, and -39.5% for Pu, *P*<0.0001); testicular TC (-46.3% for Ch and, -46.6% for Pu, *P*<0.0001); serum TG (-26.4% for Ch and -28.2% for Pu, *P*<0.0001); and serum LDL (-55.7% for Ch, and -55.2%, for Pu *P*<0.0001) respectively when compared to the untreated STZ rats. It is worth noting that the administration of Ch seed extract to diabetic animals resulted in a significantly higher serum HDL level (+59.2%, *P*<0.0001) compared to Pu seed extract (+44.6%, *P*<0.01) in comparison to the untreated STZ group.

### Oxidative stress markers profile assessment

Untreated diabetic models demonstrated a significant elevation in the testicular MDA level, AO and XO activity (+37.3%, +47.5%, +199.2%, *P*<0.0001, respectively), meantime a marked lessen in GSH content, SOD, TAC and CAT activity (-66.3%, -31.4%, -49.3%, -40.4%, *P*<0.0001, respectively) with comparing to the control group.

**Fig 4:**
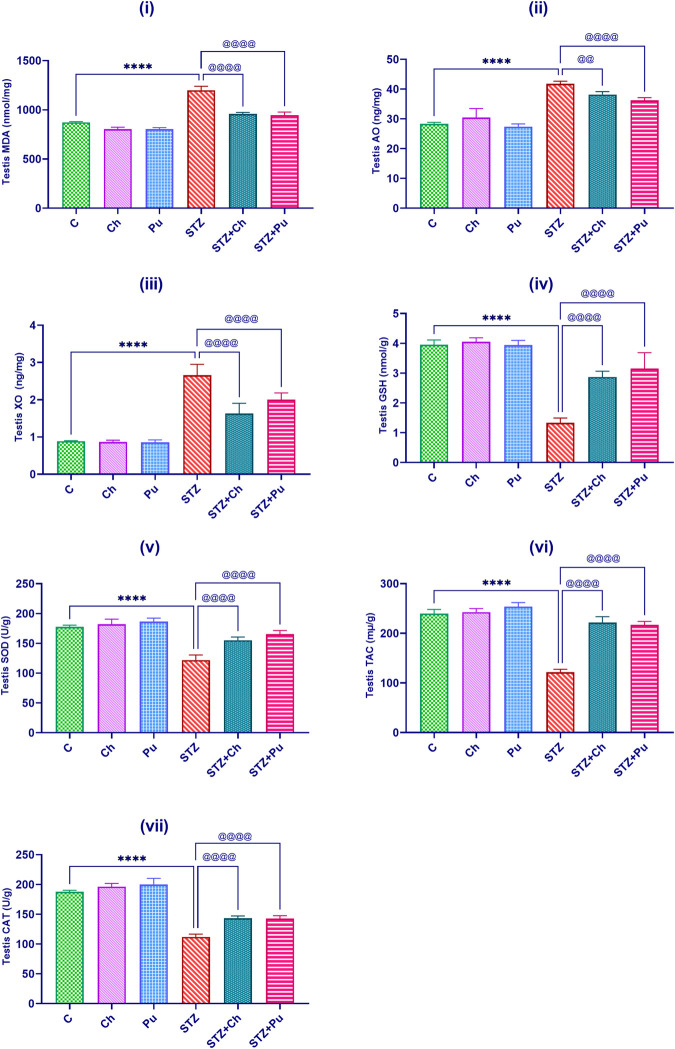
Effect of STZ, Ch and Pu on: (i) Testis MDA (nmol/mg); (ii) Testis AO (ng/mg), (iii) Testis XO (ng/mg); (iv) Testis GSH (nmol/g); (v) Testis SOD (U/g); (vi) Testis TAC; (vii) Testis CAT (U/g) in the different groups. Values are expressed as means ± SEM; (n = 6). (****P < 0.0001 vs. control group. ^@@^*P* < 0.01 and ^@@@@^*P* < 0.0001, vs. STZ group).

When compared to untreated STZ group, STZ+Ch and STZ+Pu groups markedly revealed an attenuation in MDA levels (-19.9% and -21%, *P* < 0.0001); AO activity (-8.5%, *P* < 0.01, and -13.2%, *P* < 0.0001); and XO activity (-38.7% and -24.7%, *P* < 0.0001) respectively with respect to untreated STZ rats as shown in Fig 4.

Furthermore, a significant increase in testicular GSH content (+115.1% and +136.1%, *P*<0.0001); SOD activity (+27.3%, and +35.7%, *P* <0.0001); TAC (+82.3% and +78.6%, *P* <0.0001) and CAT activity (+27.3% and +27.4%, *P* <0.0001) was detected in diabetic rats after 30 days administration of Ch and Pu seed extracts comparing with untreated STZ group as shown in Fig 4.

### Ch and Pu seed extract improved pro-inflammatory cytokines in diabetic rats

**Fig 5:**
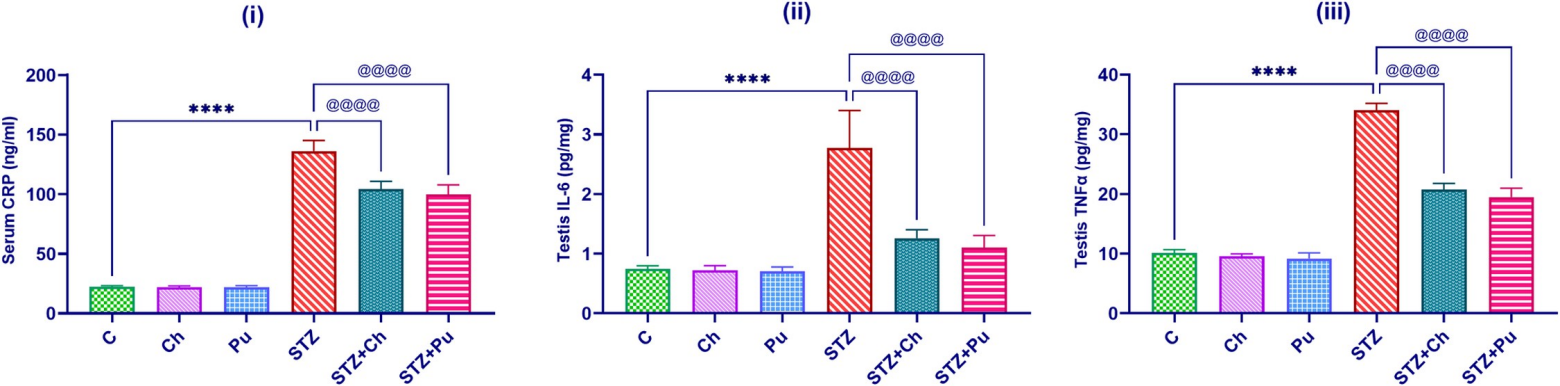
Effect of STZ, Ch and Pu on: (i) CRP (mg/dl); (ii) IL-6 (ng/ml); (iii) TNF- α (ng/ml) of different experimental groups. Values are expressed as means ± SEM; (n = 6). ****P < 0.0001 vs. control group. ^@@@@^ *P* < 0.0001 vs. STZ group.

Serum CRP level, testis IL-6 and TNF-α levels were comparable to normal control after successive 30 days of Ch and Pu seed-extracts oral injection. While the aforementioned parameters were significantly higher in the untreated diabetic rats comparing to the normal rats (+512.1%, +271.5%, +236.6%, *P* <0.0001, respectively). Otherwise, treatment of diabetic rats with Ch or Pu seed extracts recorded a substantial decline in serum CRP (-23.3 and -26.6%, *P* <0.0001); testicular IL-6 (-54.2% and -60.2%, *P* <0.0001) and testicular TNF-α (-38.9% and -42.7%, *P* <0.0001) levels, respectively with regard to the untreated diabetic rats (Fig 5).

### Ch and Pu seed extract modulated oxidative DNA damage

**Fig 6:**
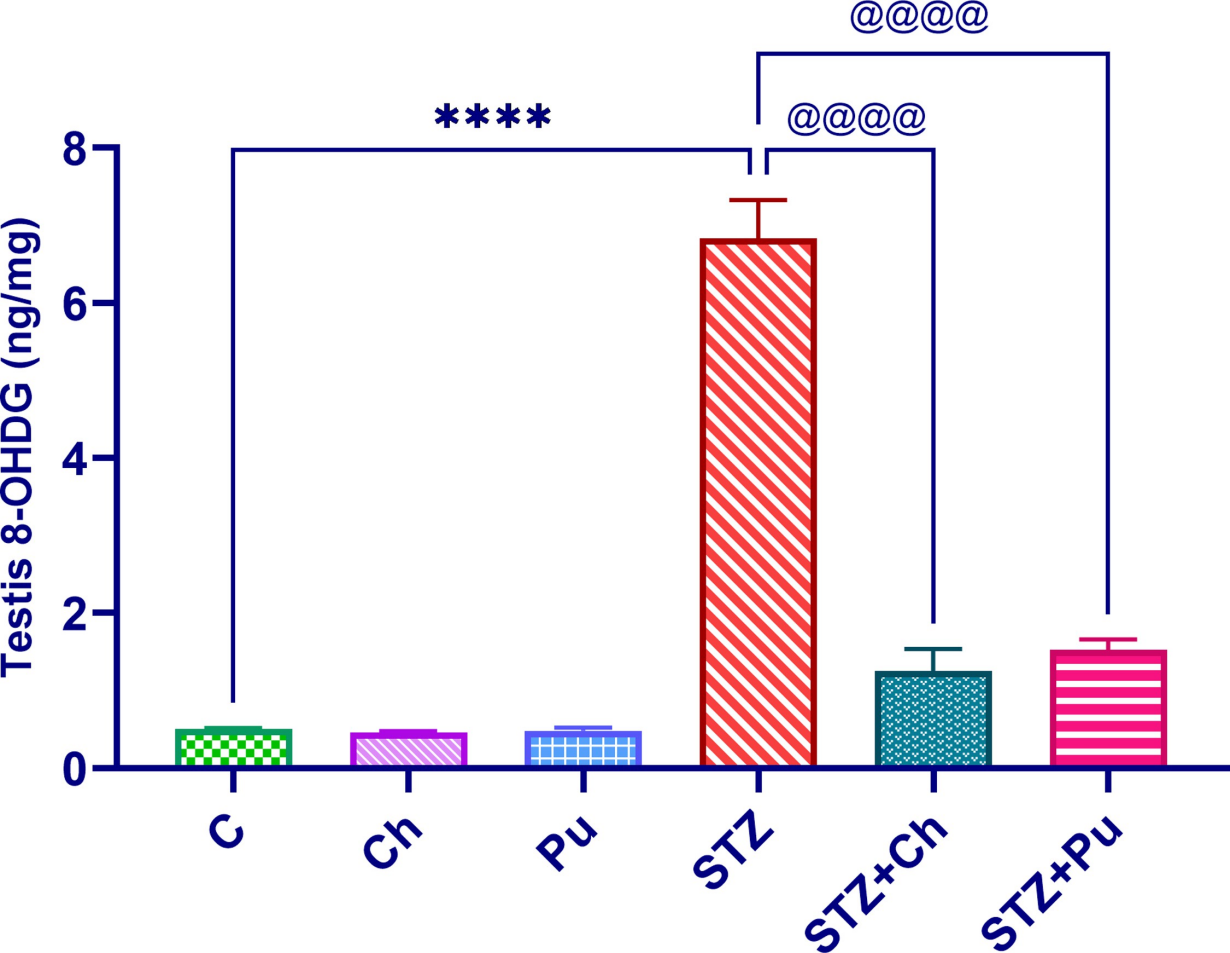
Effect of STZ, Ch and Pu on testicular 8OHdG level of different experimental groups. Values are expressed as means ± SEM; (n = 6). *P* < 0.0001 vs. control group. ^@@@@^ *P* < 0.0001 vs. STZ group.

Administration of both Ch and Pu to healthy rats resulted in a non-significant change of the testicular 8OHdG values which were comparable to those of control animals (0.46 and 0.48 ng/mg respectively). Contrary to this, testicular 8OHdG level revealed a remarkable elevation in untreated diabetic animals (+1242.9%, *P* <0.0001) respecting to normal control. It is noteworthy that the administration of Ch or Pu seed extracts to diabetic rats showed a considerable improvement in testicular 8OHdG levels (-81.7% and -77.7%, *P* <0.0001) respecting to untreated STZ group) as shown in (Fig 6).

### Pathological lesions in testes

**Fig 7:**
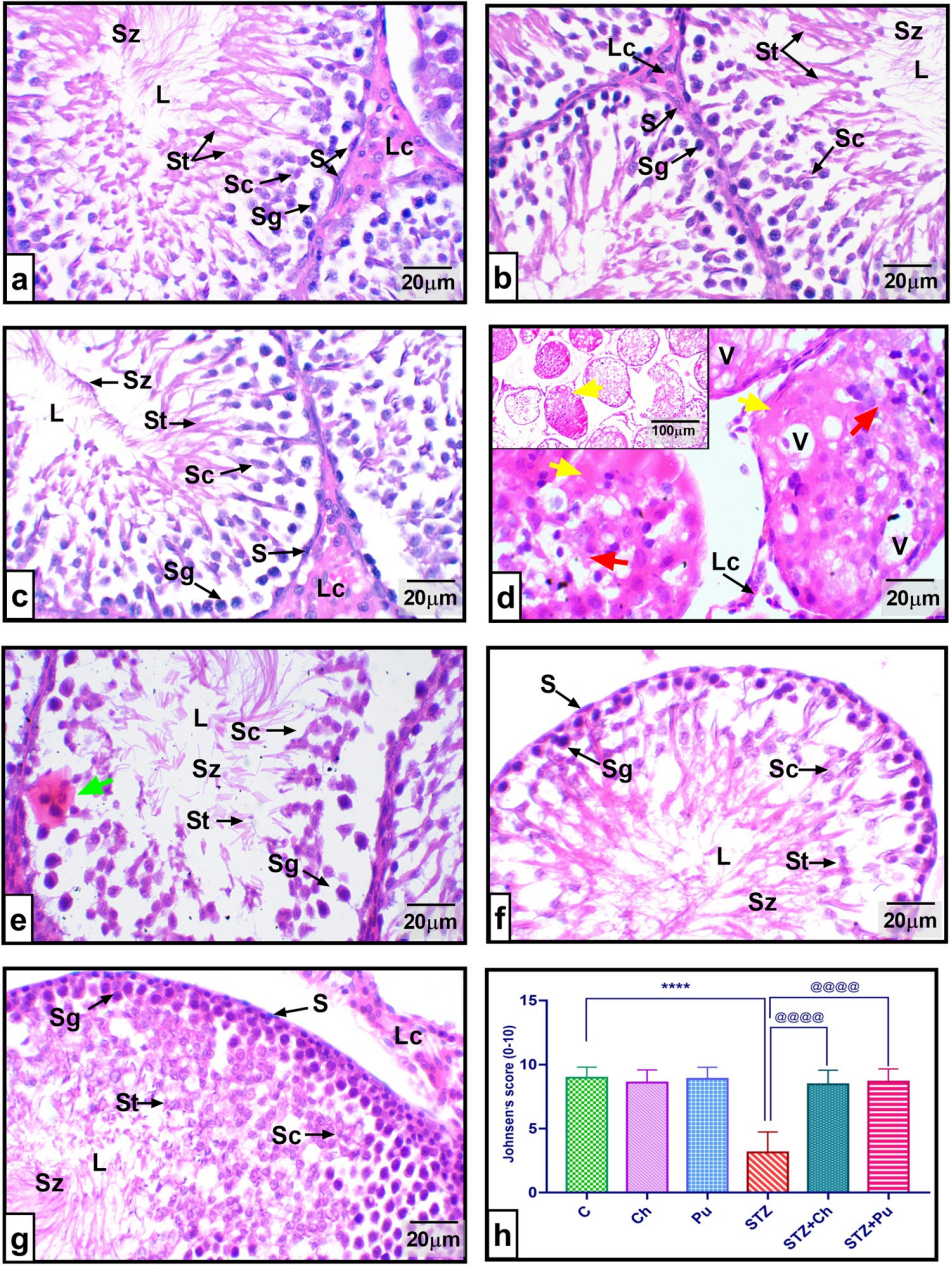
Effect of STZ, Ch and Pu on testicular histopathology: (a). Control group; (b). Ch group; (c). Pu group; Revealing typical Leydig cells (Lc), Sertoli cells (S), spermatogonia (Sg), spermatocytes (Sc), spermatids (St), and spermatozoa (Sz) in the seminiferous tubular lumen (L); (d and e). STZ group depict necrotic seminiferous tubules (yellow arrow), vacuolation (V), disorganized germinal layers (red arrow), diffused Leydig cells (Lc), Multinucleated cell (green arrow), and degenerated spermatogenesis stages (Sg, Sc, St and Sz) in the tubular lumen (L); (f). STZ+Ch group shows an almost normal spermatogenesis process; (g). STZ+Pu group displays an intact spermatogenesis process; (h). Johnsen’s score. Values are expressed as means ± SEM; (n = 6). **** *P* < 0.0001, vs. control group. ^@@@@^ *P* < 0.0001 vs. STZ group.

Testes from healthy controls (C, Ch and Pu groups) showed typical seminiferous tubules upon histopathological examination, defined by the presence of multiple tubules lined with intact Sertoli cell layers, followed with the unique stages of spermatogenesis process: spermatogonia; spermatocytes; spermatids and finally spermatozoa in the tubular lumen Fig 7A–7C. Conversely, untreated diabetic testes revealed numerous partial or segmental tubular degeneration and vacuolation owing to germ cells deficiency. Multinucleated cells are noticed within the disorganized germinal layers. Furthermore, necrotic seminiferous tubules were shown here and there within the testicular tissue characterized by excessive-eosinophilic undifferentiated cells. Leydig cells were diffused and disassembled and could also be seen Fig 7D and 7E.

Testes of diabetic rats which were given 250 mg/kg of Ch seed extract showed a partial recovery in spermatogenic cell distortion and Leydig cell deformation (Fig 7F). While supplementing diabetic rats with 200 mg/kg of Pu seed extract depicted quite normal seminiferous tubules with an adequate spermatogenesis process (Fig 7G). It is interesting to mention that the Johnsen’s scores exhibited a statistically significant increase following the administration of both Ch and Pu seed extract, in comparison to the diabetic group (*P* < 0.0001) as shown in (Fig 7H).

### Ch and Pu seed extract modulated apoptotic regulating proteins in the testes

**Fig 8:**
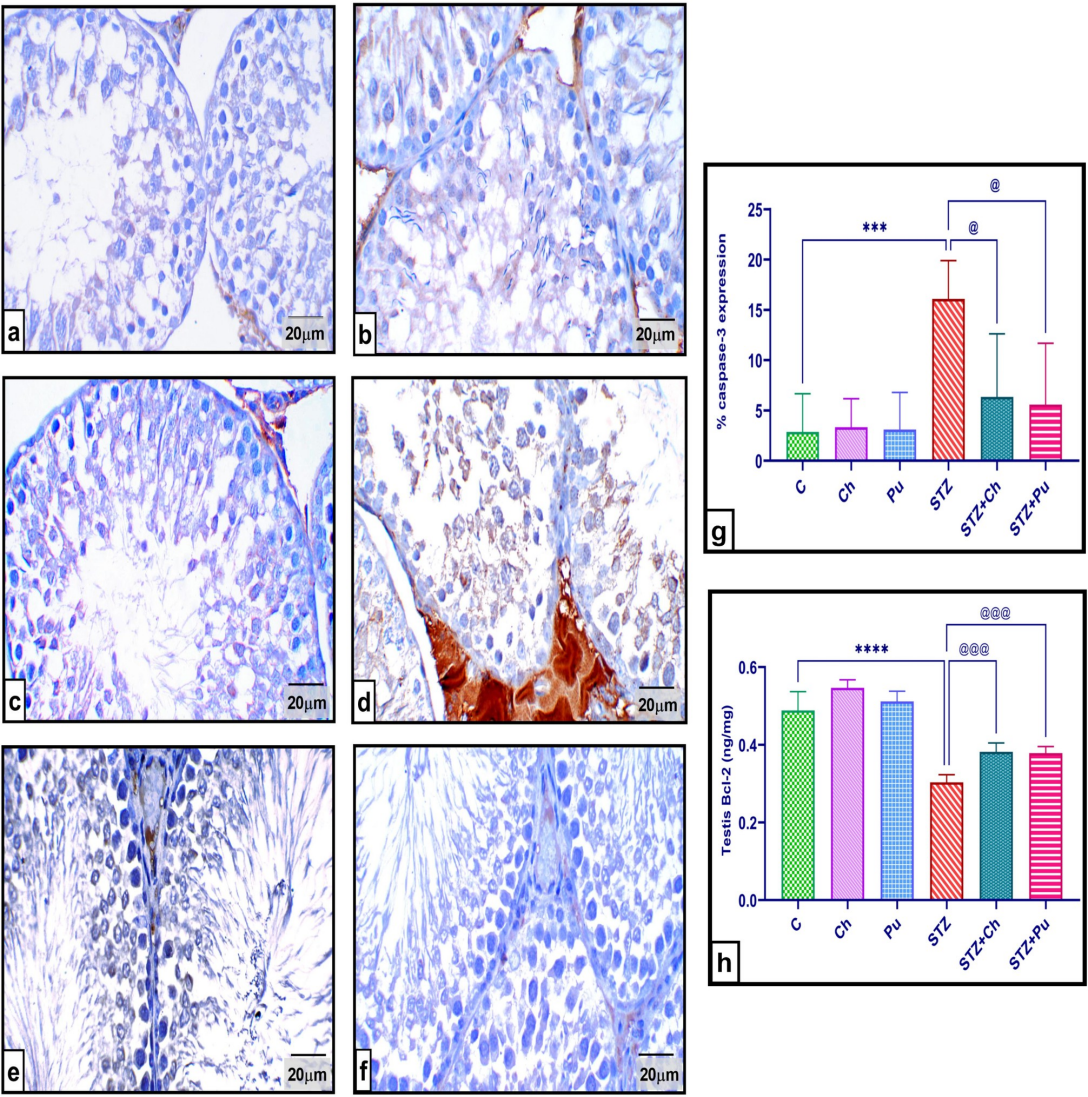
Effect of STZ, Ch and Pu on testicular caspase-3 (immunohistochemically) and Bcl-2 (ELISA) of different groups: (a). Control group; (b). Ch group; (c). Pu group; Revealing a mild caspase-3 immuno-reactivity; (d). STZ group depicts intensive expression of caspase-3; (e). STZ+Ch group shows a moderate expression of caspase-3; (g). STZ+Pu group displays caspase-3 weak expression; (h). % of caspase-3 area expression; (g). Bcl-2 testicular level. Values are expressed as means ± SEM; (n = 6 microscopic fields/tissue samples of caspase-3 immune-expression, 400X or 5 tissue samples/group). ***P < 0.001, **** *P* < 0.0001 vs. control group. ^@^*P* < 0.05, ^@@@^ *P* < 0.001 vs. STZ group.

Immunohistochemical observation of the testicular tissues from control and normal animals treated with Ch, Pu showed a mild expression of caspase-3 protein. While STZ injection significantly elevated caspase-3 expression respecting to the normal group (*P* <0.001) and showed an intensive protein expression in the tissue. Same time, Oral dosing of Ch, Pu extract after diabetes induction significantly regressed caspase-3 level (-60.5% and -65.2%, *P* <0.05) respectively, with respect to the untreated diabetic rats, and normalized its tissue expression (Fig 8A–8G).

Meantime, STZ injection resulted in a statistically significant (*P* < 0.0001) decline in the testicular levels of the anti-apoptotic protein Bcl-2 comparing to normal control. Otherwise, treatment of diabetic rats with Ch or Pu seed extracts significantly ameliorated testicular Bcl-2 level (+25.8 and +24.7 ng/mg, *P* < 0.0001) successively with respect to untreated diabetic rats (Fig 8H).

### Ch and Pu seed extract enhanced vimentin expression in the testes

**Fig 9:**
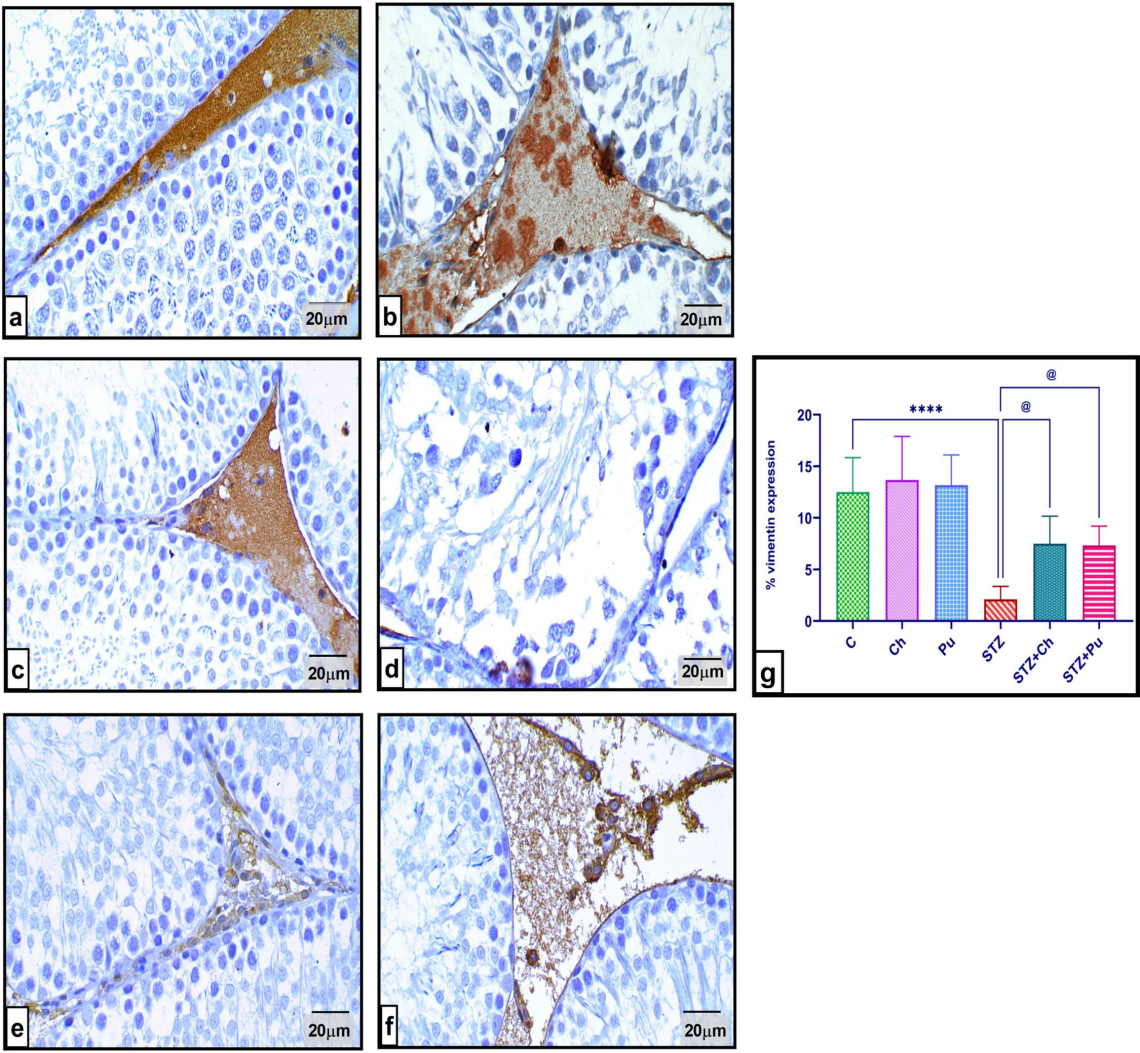
Effect of STZ, Ch and Pu on testicular vimentin (immunohistochemically) of different groups: (a). Control group; (b). Ch group; (c). Pu group; Revealing an intensive vimentin immuno-reactivity; (d). STZ group depicts a weak expression of vimentin; (e). STZ+Ch group shows a moderate expression of vimentin; (f). STZ+Pu group displays a mild vimentin expression; (g). % of vimentin area expression. Values are expressed as means ± SEM; (n = 6 microscopic fields/tissue samples of vimentin immune-expression, 400X, 5 tissue samples/group). **** *P* < 0.0001 vs. control group. ^@^ *P* < 0.05 vs. STZ group.

Immunohistochemical observation of testicular tissue from normal animals (C, Ch, and Pu groups) revealed intensive vimentin expression. While diabetic group showed weak vimentin immunoreaction within the destructed seminiferous tubules. Otherwise, treating the diabetic rats with Ch and Pu attuned the vimentin expression (*P* <0.05 comparing to STZ group), as shown in (Fig 9).

### Ch and Pu seed extract significantly mitigated testicular malfunction accompanied with DM

**Fig 10:**
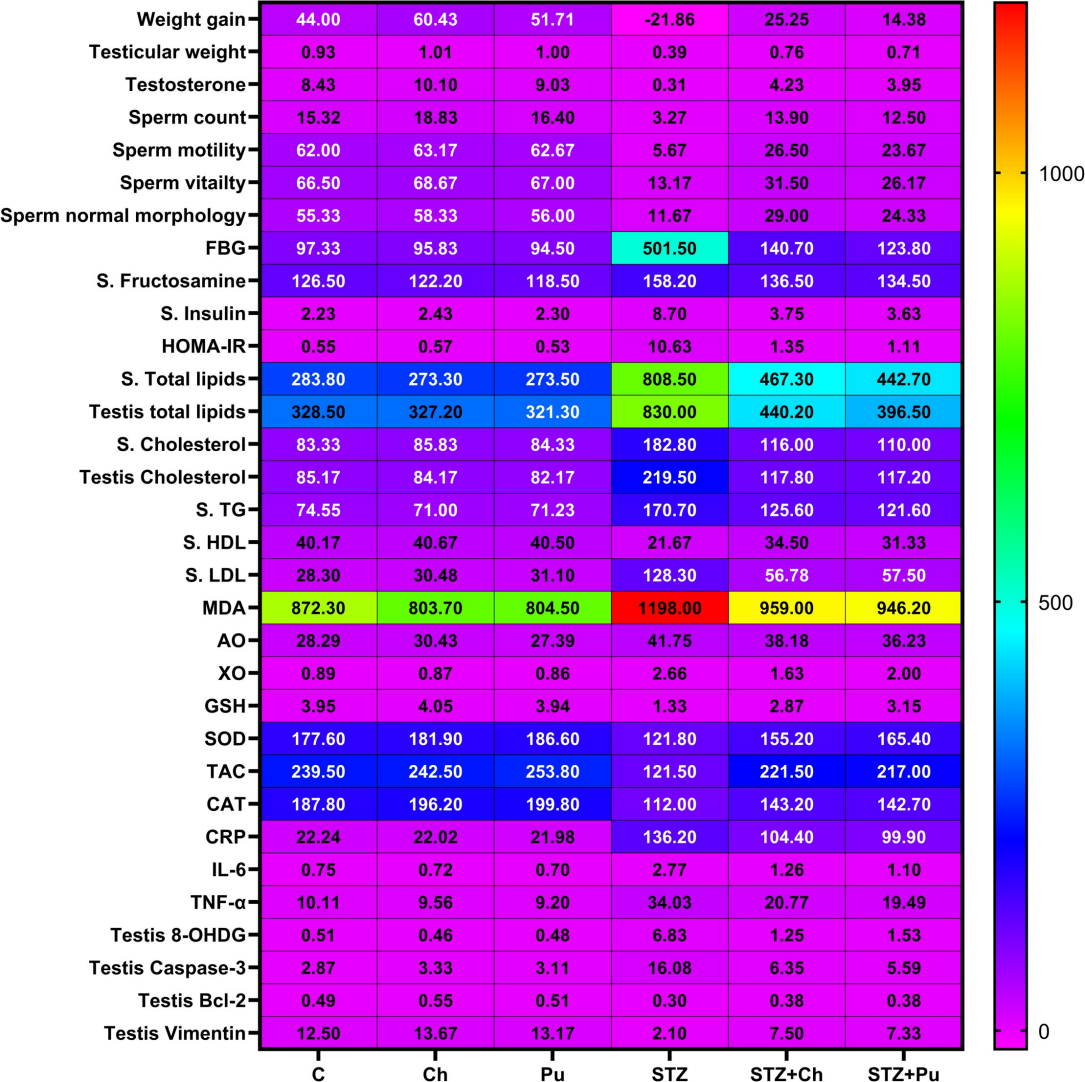
Concluded results of the study represented via heat map.

Observing Fig 10, it is evident that treating diabetic rats with either Ch or Pu resulted in considerable improvement in several testicular indicators, particularly in terms of inflammatory oxidative stress, histological architecture, and apoptotic state.

## Discussion

Chronic hyperglycemia known as DM is associated with several organs and systems failure, including the reproductive system [52]. In the current investigation, the treatment of rats with STZ led to a notable reduction in pancreatic β-cells, hence causing an increase in glucose levels that subsequently triggered the release of insulin and the development of insulin resistance, both of these are prominent characteristics of T2DM [53]. Persistent hyperglycemia has been linked to elevated reactive oxygen species production and significant cellular harm [54]. The rats with diabetes displayed increased levels of oxidative stress, resulting in negative impacts on the integrity of testicular DNA, reduction in sperm count, and impairment of spermatogenesis, finally resulting in infertility [55]. Moreover, ROS possess the capability to disrupt cellular antioxidant defense mechanisms or trigger inflammatory signaling pathways, finally resulting in testicular apoptosis [56].

There are now oral hypoglycemic medications that are accessible for diabetes treatment. However, there is a growing inclination towards the utilization of herbal treatment as a result of the adverse consequences associated with conventional pharmaceuticals [57]. A dietary regimen that is abundant in antioxidants has been shown to significantly decrease the incidence of several diseases associated with oxidative stress. Plant phenolics are bioactive compounds found in plants that are important components of the human diet. These secondary metabolites possess antioxidant characteristics and have been shown to contribute to the promotion of human health. The compounds have high intestinal absorption rates, accompanied by minimal toxicity and cost-effectiveness [58, 59]. The present work demonstrates the potential hypoglycemic and protective benefits of extracts derived from Ch and purslane seeds in relation to diabetes and its associated testicular problems. The observed benefits can be ascribed to the existence of phenolic compounds and other antioxidant properties present in these extracts.

The experimental findings demonstrated a significant decrease in blood glucose levels, serum insulin levels, insulin resistance, and fructosamine levels in diabetic rats subjected to treatment with Ch and Pu. The potential mechanism by which Ch and Pu exert their hypoglycemic effects may involve the facilitation of glucose transportation to peripheral tissues, or maybe through other processes, for instance, the activation of glucose absorption by peripheral tissues [60]. The potential of the ethanolic extract of Ch to mitigate DM is demonstrated through its ability to decrease hepatic glucose-6-phosphatase activity and enhance insulin resistance [61].

The heightened oxidative stress seen in this study may be attributed to the ongoing production of ROS caused by hyperglycemia and glucose autoxidation [62]. Oxidative stress is a condition characterized by an imbalance between the production of harmful free radicals and the ability of the body to neutralize them via the use of antioxidants [63]. In this work, we assessed the extent of oxidative damage in rats with diabetes caused by STZ. This was done by examining lipid peroxidation end-product (MDA) level, as well as the activities of AO and XO enzymes. Our findings indicated an increase in the expression of AO and XO enzymes, suggesting an elevation of oxidative damage. Additionally, we observed a notable reduction in the concentrations of antioxidants in the testicular tissues of these rats.

This finding aligns with a recent study which documented that STZ therapy resulted in a notable elevation in the concentration of MDA. Lipid peroxidation represents a prominent physiological response arising from the condition of oxidative stress. MDA is a well-established byproduct resulting from the secondary process of lipid peroxidation. This compound holds significant recognition and can be effectively employed as a tool for the detection and characterization of cellular membrane impairment [19]. In addition, the body’s antioxidant defense capacity, including glutathione, glutathione peroxidase, and glutathione S-transferase, is capable of effectively neutralizing free radicals. Glutathione plays a crucial role in combating lipid peroxidation [64].

Additionally, the occurrence of hyperglycemia, elevated the levels of insulin and leptin also contribute to the induction of oxidative stress [65]. The testes experience heightened oxidative stress due to hyperglycemia, as evidenced by earlier research. This phenomenon may perhaps account for the elevated prevalence of infertility among diabetic males [66]. The auto-oxidation of glucose and non-enzymatic glycation results in an increase in the production of hydrogen peroxide and superoxide radical (O2). This increase in reactive oxygen species negatively impacts the activity of key antioxidative enzymes, namely peroxidase, catalase, superoxide dismutase, and glutathione S-transferase (GST), in individuals with diabetes [67]. The reduced enzymatic activity of SOD leads to an increase in the generation of molecular oxygen (O2) within the specific tissues [68]. A reduction in the activity of peroxidase leads to a diminished capacity to defend against ROS [69, 70]. The overproduction ROS leads to an imbalance in the cellular redox equilibrium, resulting in an oxidative state that is associated with various male reproductive issues [17, 71].

The mitigation of oxidative stress in this present study was achieved through the administration of extracts derived from Ch and Pu seeds. The antioxidant properties exhibited by Ch are attributed to its abundant concentration of phenolic chemicals and flavonoids [72]. The antioxidant activity of the phenolic content can be attributed to the presence of hydroxyl groups, which demonstrate the ability to scavenge free radicals [73]. In a prior experiment, DM induced a significant elevation in blood glucose, protein carbonyl content and lipid peroxidation which was accompanied with declined CAT and GSH level and neuronal activity impairment in cerebral hemispheres of albino rats, 48 h after alloxan monohydrate administration. Meantime, feeding these rats on dried powder leaves of Ch decreased blood glucose levels to near-normal levels and played a vital part in the augmentation of the endogenous antioxidant defense system through the mitigation of oxidative stress, restoration of GSH content, and enhancement of CAT [74]. Another study aimed to evaluate the effects of enriched Ch inulin supplementation on glucose homeostasis, liver enzymes, serum calcium and phosphorous concentrations and hematological parameters in female patients with T2DM receiving a daily dose of 10 g of Ch, results showed significant reductions in FBG, insulin, HbA1c, AST and ALP concentrations. [75] Furthermore, Ch inulin has demonstrated a significant therapeutic impact on metabolic disorders throughout its extensive historical usage [76, 77]. The defense mechanism of Pu is believed to arise from its capacity to scavenge free radicals, which are recognized for their role in inducing oxidative harm to lipids, proteins, and nucleic acids [78]. The current investigation has documented the presence of properties associated with free radical scavenging and autoxidation in Ch and Pu. The capacity of these traits to decrease the concentrations of MDA and the activity of SOD as well as CAT, is evident. Moreover, it has been observed that Ch and Pu have the ability to replenish TAC and GSH levels. The observed results could perhaps be attributed to the existence of antioxidant substances, including anthocyanins, flavonoids, polyphenols, and vitamin C, which may have a main role in safeguarding against formation of free radicals [79–81].

The present investigation attributes the observed testicular dysfunction, characterized by diminished sperm parameters and testosterone hormone levels, to the presence of oxidative stress and an excessive production of ROS [82, 83]. The presence of hyperglycemia in individuals with T2DM exerts an adverse influence on testicular function. The observed effect encompass reduction in the number of spermatogonia, Sertoli cells, and Leydig cells, leading to a decline in the process of sperm generation [84].

Semen analysis encompasses a series of examinations that assess the functionality of the male reproductive organs and systems, serving as an indicator of sperm quality [85]. The assessment of testicular function is conducted through the measurement of sperm concentration, whereas the evaluation of the toxic effects resulting from exposures to sperm cells is performed by examining sperm morphology [86, 87]. The current investigation has demonstrated that hyperglycemia generated by diabetes is associated with diminished quality of sperm, as evidenced by a reduction in sperm count, motility, vitality, and normal morphology. The results of this study are in consistent with prior studies which reported that Diabetic patients exhibit spermatozoa characterized by heightened DNA fragmentation, as well as an elevation in advanced glycation end products (AGEs) and their corresponding receptors [88, 89]. These factors all contribute to the decline in sperm quality, impaired sperm functions, alterations in testicular metabolite levels, and modifications in genes expression involved in spermatogenesis [88].

Spermatozoa are susceptible to oxidative stress caused by ROS as a result of their elevated levels of polyunsaturated fatty acids (PUFA) and abundance of mitochondria. Hence, Spermatozoa exhibit heightened susceptibility to oxidative stress, a condition that can induce lipid peroxidation, facilitate peroxidative harm, impair sperm motility, disturb Leydig cells responsible for testosterone production, and elevate oxidative DNA damage [90–92].

The hormone testosterone (T), produced by Leydig cells in the testes, is responsible for regulating gene expression associated with sexual function, hence playing a significant role in male reproductive processes. T has an essential role in facilitating the development of male puberty, supporting spermatogenesis, and ensuring the preservation of secondary sexual characteristics [93]. Additionally, it exerts regulatory control over the secretory functions of the accessory glands, hence modulating seminal fructose levels [16, 94]. As a result, a decrease in testosterone levels leads to a reduction in fructose concentration within semen, so affecting the typical motility of sperm.

Results in the current investigation revealed that, the treatment of Ch and Pu resulted in a decrease in testicular anomalies and histological alterations in diabetic rats as compared to untreated diabetic rats. The administration of Ch and Pu effectively mitigated testicular dysfunction by elevating serum T level and enhancing indicators of spermatogenesis, such as sperm count, motility, and morphological index. Pu extract demonstrated a significant enhancement in the histological alterations observed in the testicular tissue, as well as an increase in the seminiferous tubules’ diameter; The findings presented align with other research that has shown the potential of Pu extracts to confer a protective impact on sperm total count and T levels in male rats [95]. The observed ability for Ch and Pu to improve testicular dysfunction can be related to the presence of flavonoid compounds, minerals, and vitamins that raise antioxidant levels and exhibit a positive influence on male reproductive function [52].

Elevated serum lipid levels reported in this study can be attributed to the unimpeded activity of lipolytic hormones on adipose tissue. Elevated levels of cholesterol and triglycerides were previously seen in rats with diabetes caused by STZ [96]. T2DM induced rats exhibit disorders pertaining to lipid metabolism, as indicated by high triglycerides and total cholesterol levels in their serum. This metabolic profile bears resemblance to that observed in humans with type 2 DM [97]. Hypercholesterolemia, a medical disorder characterized by elevated levels of cholesterol in the bloodstream, has been found to potentially initiate an inflammatory response, resulting in an upregulation of pro-inflammatory cytokines such as TNF-α and IL-6. Consequently, this phenomenon can intensify the presence of testicular oxidative stress, thereby leading to potential tissue impairment [98].

The present study documented the hypolipidemic impact of extracts derived from Ch and purslane seeds, specifically in terms of reducing TL, TC, LDL-C and increasing HDL-C in rats with DM. The hypocholesterolemic action of Ch can potentially be ascribed to the presence of isoflavones within this botanical species. It is widely hypothesized that isoflavones possess the ability to impede the process of cholesterol absorption in the intestines by competitive binding to absorption sites. Consequently, the contents of the jejunum exhibit elevated levels of unabsorbable cholesterol, while faecal materials display increased amounts of cholesterol [88]. Pu also showed a significant reduction in plasma triglyceride and LDL-cholesterol levels, while concurrently elevation of HDL-cholesterol levels in the bloodstream [38].

The present investigation exhibited a significant increase in inflammatory biomarkers, including TNF-α, IL-6, and CRP. The pathogenesis of T2DM is significantly influenced by the presence of inflammation [99]. This observation implies a clear association between hyperglycemia and the attendance of markers indicating inflammation in rats with diabetes. Under normal physiological settings, the presence of TNF-α in the testis is minimal. However, prior studies have indicated that TNF-α expression in testis is increased in different stages of reproductive failure [100].

Ch displays various therapeutic effects on persons with type 2 DM, including anti-diabetic, antihypertriglyceridemic, anti-oxidative, and anti-inflammatory qualities [79]. The potential anti-inflammatory properties of Ch are attributed to its role in downregulation of the expression of proinflammatory cytokines, such as TNF-α. TNF-α is known to be synthesized during the initial phases of inflammation and plays a crucial role in regulating the release of other cytokines [101]. Pu also revealed the capacity to elevate vascular endothelial growth factor (VEGF) levels and reverse the elevated TNF-α levels generated by streptozotocin in testicular tissue [102]. A further investigation demonstrated the antioxidant properties of Pu, resulting in a notable reduction of TNF-α and IL-6 levels in diabetic rats [103].

The observed elevation in apoptosis within the context of this investigation may be attributed to the excessive presence of oxidative stress commonly associated with DM. The maintenance of sperm quality and quantity in normal testicular tissue is achieved by the process of spontaneous apoptosis, which involves the elimination of damaged or defective germ cells. However, the dysregulation or excessive occurrence of apoptosis may lead to the demise of testicular cells, ultimately resulting in impaired spermatogenesis and infertility [104]. Male rats with T2DM exhibited regressive alterations in spermatogenesis, an elevated count of apoptotic germ cells, and reduced synthesis of testosterone. These findings indicate a potential impairment in reproductive capabilities among DM male rats. The findings of this study align with prior research that has shown the occurrence of hypogonadism in patients diagnosed with DM [105]. The reduction of spermatogenesis in rats with DM is attributed to a dysfunction in the activity of Sertoli cells. These cells are accountable for the secretion of androgen-binding protein, which serves a pivotal function in the maintenance of testosterone levels within the seminiferous tubules [106, 107].

Occurrence of apoptosis induced by hyperglycemia leads to the detrimental effects on specific organs [108, 109]. The impact of hyperglycemia, oxidative stress, and microcirculation disturbance on apoptosis has been reported to be substantial [110]. The plasma membrane of the sperm cell has a significant presence of PUFA, making it prone to oxidative harm induced by ROS [111, 112]. Reactive oxygen species have been found to initiate lipid peroxidation of the sperm membrane, hence promoting alterations in membrane fluidity that ultimately lead to a decrease in sperm motility [113, 114]. Additionally, it has been observed that this phenomenon also affects the mitochondrial and nuclear DNA, resulting in an increased level of fragmentation in sperm DNA [115] and apoptosis [116, 117].

The most prevalent DNA aberration linked to suboptimal sperm quality and reduced rates of fertilization is DNA fragmentation [118]. Sperm DNA fragmentation often arises from two primary factors, namely oxidative DNA damage and apoptosis [119, 120]. The present investigation observed a notable elevation in DNA fragmentation marker (8OHdG). This was accompanied by a considerable rise in caspase 3-mediated apoptosis and a drop in Bcl-2 expression, as well as a reduction in vimentin expression, among diabetic rats. Meanwhile, when the DM rats were administered Ch and Pu seed extracts for a period of 30 days, testicular dysfunction was improved through the acquisition of notably reduced amounts of 8OHdG and caspase 3, together with an enhanced Bcl-2 level and percentage of vimentin expression.

The ability of Ch and Pu to suppress the initiation of apoptotic cell death and facilitate the progression of the cell cycle lies in their antioxidant characteristics. Ch has been found to be both safe and effective in enhancing the oxidative condition of the testes, promoting androgenic activity, and supporting the process of spermatogenesis [52]. Moreover, Pu exhibits notable efficacy as an antioxidant and hypoglycemic mediator [121]. The utilization of Pu as a therapeutic intervention demonstrated efficacy in modulating apoptosis and cell proliferation within the testes by downregulating the expression of caspase-3. The results of this study were significantly corroborated by the therapeutic effects of Pu on histological testicular abnormalities [54].

## Conclusions

In summary, the administration of Ch and Pu seed extracts has been found to improve the body’s antioxidant defense system, leading to a reduction in hyperglycemia, hyperlipidemia, oxidative stress, inflammation, and apoptotic pathways associated with T2DM. Ch and Pu seed extracts have been found to offer protection against testicular dysfunction generated by DM. This protective effect is observed through their ability to improve sperms and testicular parameters and reduce DNA fragmentation.

## Supporting information

S1 File (XLSX)
